# Serial dependence tracks objects and scenes in parallel and independently

**DOI:** 10.1167/jov.22.7.4

**Published:** 2022-06-10

**Authors:** Thérèse Collins

**Affiliations:** 1 Integrative Neuroscience and Cognition Center, University of Paris and CNRS, Paris, France

**Keywords:** serial dependence, perception, history effects

## Abstract

The visual world is made up of objects and scenes. Object perception requires both discriminating an individual object from others and binding together different perceptual samples of that object across time. Such binding manifests by serial dependence, the attraction of the current perception of a visual attribute toward values of that attribute seen in the recent past. Scene perception is subserved by global mechanisms such as ensemble perception, the rapid extraction of the average feature value of a group of objects. The current study examined to what extent the perception of single objects in multi-object scenes depended on previous feature values of that object or on the average previous attribute of all objects in the ensemble. Results show that serial dependence occurs independently on two simultaneously present objects, that ensemble perception depends only on previous ensembles, and that serial dependence of an individual object occurs only for the features of that particular object. These results suggest that the temporal integration of successive perceptual samples operates simultaneously at independent levels of visual processing.

## Introduction

The visual world as we perceive it is made up of objects and scenes. Perceiving individual objects requires both discriminating them from others and properly binding together different perceptual samples of the same object across viewpoints, retinal locations, changes in ambient lighting, and time. Temporal binding is thought to be subserved by a mechanism referred to as a continuity field, a spatiotemporally tuned operator that integrates successive perceptual samples over a brief moment in time and across a restricted region of space. Continuity fields may promote perceptual stability across time by smoothing out spurious fluctuations in the proximal stimulus. Continuity fields can be measured in the laboratory as a phenomenon referred to as serial dependence, small misperceptions of currently presented visual attributes as pulled toward values of that attribute seen in the immediate past.

Perceiving scenes is aided by mechanisms that extract summary statistics and average them into global representations of cluttered environments (Alvarez, 2011). Observers are remarkably good at extracting the average value of a group of objects, such as the average orientation of a group of Gabor patches, the average speed and direction of moving dots, or the average emotional expression in a crowd of faces (e.g., Haberman & Whitney, 2007). The attributes of the individual objects that make up a group are often less well perceived than the average attribute (Ariely, 2001). Furthermore, the extraction of average attributes occurs for stimuli even in the absence of an explicit task, as shown, for example, by the influence of distractor summary statistics on target search performance (Hansmann-Roth, Kristjánsson, Whitney, & Chetverikov, 2021).

The two effects may be contradictory: the attraction of object perception toward feature values in its recent past versus the perceptual predominance of the average attribute. It remains unknown how individual object versus ensemble representations are integrated across time.

Serial dependence has been examined mainly in the context of sequential presentations of a single object and observer reports of a feature of that object. It is thus unknown to what extent the perception of individual objects in multi-object scenes depends on previous ensemble representations of the scene or whether independent representations can be maintained for individual objects and global scene characteristics. Ensemble perception is subject to serial dependence. When asked to report the average attribute of a set of stimuli, subject responses were pulled toward the average of the previous set (Manassi, Liberman, Chaney, & Whitney, 2017; Pascucci, Mancuso, Santandrea, Della Libera, Plomp, & Chelazzi, 2019). Manassi et al. (2017) also showed that serial dependence occurred between single objects and ensembles, and vice versa, but they did not contrast the effect of a previous individual object versus a previous ensemble on current perception. This is the crucial question to determine how object and scene representations interact to ensure perceptual stability across time.

The main question of interest here is whether an individual object that has been averaged into an ensemble representation can still influence upcoming perception of the individual item. If that were the case, it would suggest that multiple continuity fields are maintained simultaneously for objects and for the ensemble representation including those objects. This seems unlikely, given the literature on how representations of individual objects in visual short-term memory is biased by the summary statistics of the current scene (Brady & Alvarez, 2011; Lew & Vul, 2015). Furthermore, long-term memories may resemble ensemble representations (Richards, Xia, Santoro, Husse, Woodin, Josselyn, & Frankland, 2014), and scene information is a powerful cue for long-term object recall (e.g., Brady, Konkle, & Alvarez, 2011; Hollingworth, 2006). It thus seems likely that individual objects that have been integrated into an ensemble representation would no longer exert an effect on subsequent individual objects. The current study examined to what extent the current perception of a single shape and the current perception of the average of a set of shapes depended on previously seen shapes or ensembles of shapes. The previous literature on ensemble perception and its influence on memory for individual objects suggests the following hypothesis: Both the current perception of an individual shape within a scene and the current perception of the average shape will be pulled toward the average shape of previous scenes.

## Materials and methods

### Participants

Fifteen subjects participated in Experiment 1 (two women; mean age, 29 years; range, 18–65). Nineteen subjects participated in Experiment 2 (three women; mean age, 33 years; range, 18–56). The experiments were run on the online platform testable.org, and subjects were recruited through the Testable Minds subject pool. The number of subjects was determined by a power analysis based on reports of serial dependence effect sizes in the literature, which places the number at around five subjects for a power of 99% (i.e., estimated 1% probability of a false negative). The number of subjects was increased because it was practically feasible and ensured greater power, given that some of the conditions tested here were expected to have smaller serial dependence than what has been reported before. The relevant ethical information pertaining to the laboratory version of the study and approved by the French national ethics committee (CPP) in accordance with the tenets of the Declaration of Helsinki was provided at the onset of the experiment, and participants had to click to accept the consent form before proceeding with the experiment. Sex, age, and screen characteristics, but no personally identifying information, were recorded for each participant.

### Stimuli

Because the experiment was run on an online platform, exact control of stimulus size was not possible. Testable.org displays stimuli as images centered on observers’ personal computer screens, adjusted for each participant's screen resolution but without distorting the image proportions (i.e., the same adjustment in width and height). The adjustment was achieved by having participants perform a brief calibration procedure at the beginning of the experiment, which consists adjusting a line to the size of a credit card (which has a fixed international standard width of 8.56 cm or 3.37 inches). Because the length of the line in pixels was known, this allowed a rough estimate of individual screen pixels per inch. This was then used to scale the images such that the physical size of the image stayed constant between screens. Observers were instructed to sit 60 cm (arm's length) from their screen for the duration of the experiment. All stimuli were 1024 × 768-pixel images; stimulus sizes below are given for this distance, but the online set-up meant that there was some variability in stimulus size. Stimuli were generated using Psychtoolbox for MATLAB (MathWorks, Natick, MA) (Brainard, 1997; Kleiner, Brainard, Pelli, Ingling, Murray, & Broussard, 2007; Pelli, 1997) and saved as images.

A black dot, 0.25 degrees of visual angle (dva) in diameter and presented at screen center, served as a fixation point. Test stimuli were 100 geometrical shapes that ranged from a circle to a square. For trials with two shapes, there were thus 10^4^ possible combinations; a random subset of 1000 trials was selected for Experiment 1 and another random subset of 1800 trials for Experiment 2. Shapes were 2.7 dva wide (circle diameter or square side). The transition from one shape to the other was made by placing, at each corner of a square, arcs defined by a central angle from 45° (circle) to 0° (square), in steps of 0.45°. For ease of interpretation, each shape morph was given a value from 0 (circle) to 1 (square); the fully ambiguous shape (value of 0.50) was not included because there is no correct response. Shapes were embedded in pixel noise. Six examples of the shape morphs can be seen in Figure 2. Masks were screens of pixel noise alone.

### Procedure

**Figure 1.**
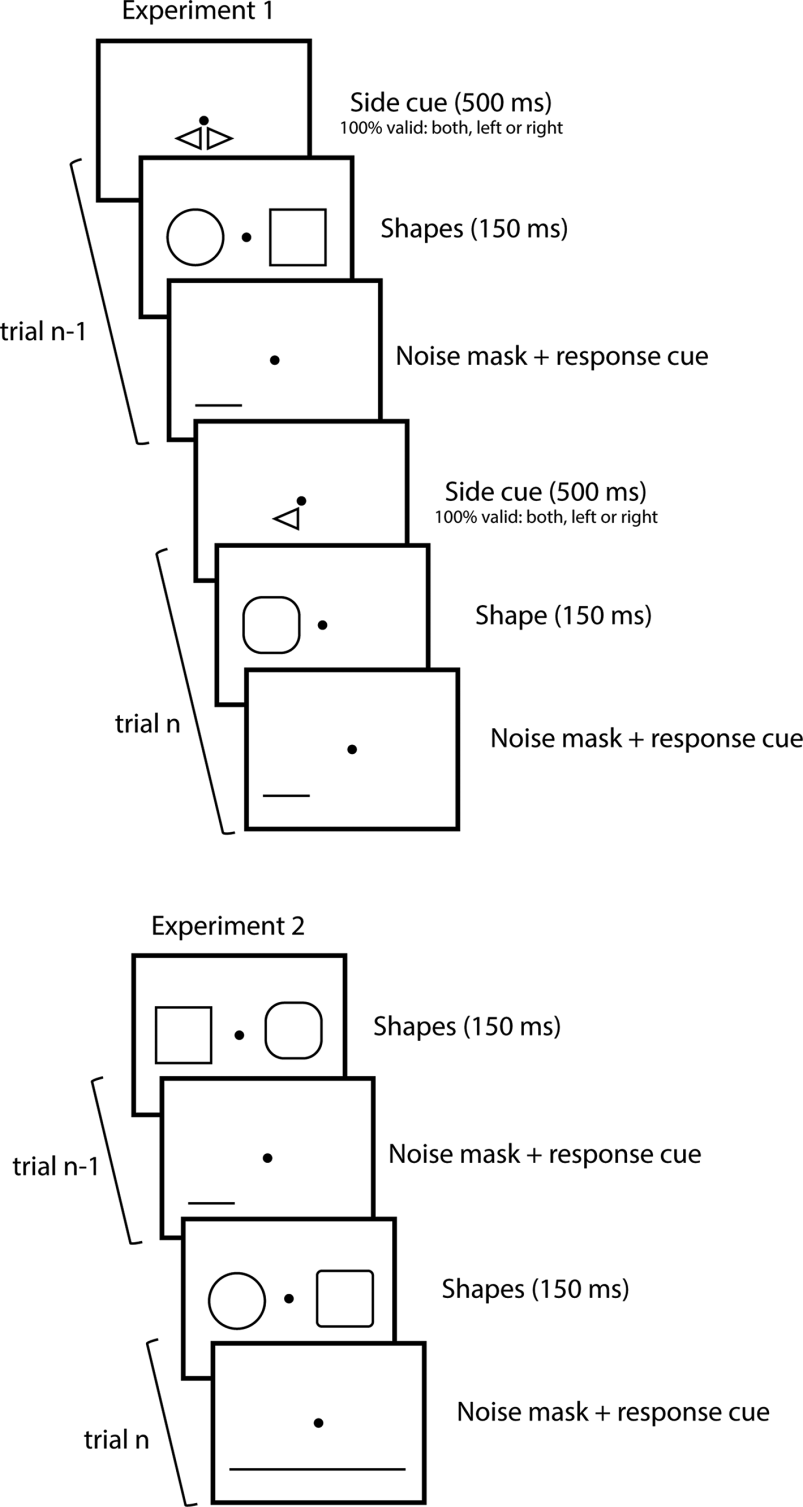
Procedure. (Top panel) Experiment 1: Two trials are pictured. In each, a 500-ms cue indicates number of shapes (one or two) and, in the case of one shape, its side (left or right). Shapes are then presented for 150 ms, followed by a noise mask and response cue until subject response. The task was to report whether they had seen a circle or a square by pressing on one of two keyboard buttons. (Bottom panel) Experiment 2: There were always two shapes, presented for 150 ms, followed by the noise mask and response cue (left, right, or average) until subject response.

Experiment 1 had two parts. The first 400 trials of the experiment were single-shape trials. On each trial, a shape appeared at screen center for 150 ms, followed by a pixel-noise mask that also served as the response screen (Figure 1). The second part of the experiment was the double-shape session, composed of 1000 double-shape trials and 400 single-shape trials (200 shapes on the left, 200 shapes on the right), randomly interleaved. The subject was cued about the number of shapes and, if there was only one, about its location (left or right). The cue was to ensure that there was no uncertainty about target location or reduced attention to the target (the strength of serial dependence decreases in the absence of attention Fischer & Whitney, 2014). In double-shape trials, two shapes appeared to the left and right of fixation for 150 ms, followed by the mask. A response cue appeared simultaneously with the mask and indicated the shape the subject was to report (left or right). Half of the trials called for a response about the shape on the left, half about the shape on the right. Subjects responded by pressing one of two buttons on the keyboard (one for “circle” and another for “square”), followed immediately by the cue for the next trial. In the interleaved single-shape trials, the procedure was identical except that only one shape appeared, on the left or right, and the response cue was always valid.

Experiment 2 was similar except that there were always two shapes and, in addition to reporting one of the two shapes (identically to the double-shape trials in Experiment 1), on most trials participants had to report the average of the two shapes. In this case, the response cue underlined both shapes. There was no pre-cue, as there were always two shapes on screen. There were 516 single-report trials (29%) and 1284 ensemble-report trials, randomly interleaved.

### Data analysis

Serial dependence was calculated in several ways:

1. By fitting psychometric functions to the proportion of “square” responses as a function of current shape, separately for trials preceded by more circular shapes and trials preceded by more square shapes. Functions were fitted using the psignifit toolbox version 2.5.6 for MATLAB which implements the maximum-likelihood method described by Wichmann and Hill (2001). Functions were fit to individual data; the average psychometric function was plotted for presentation purposes by averaging parameters obtained on individual fits. Two parameters are of interest: the slope of the psychometric function and the point of subjective equality (PSE)—that is, the stimulus level for which subjects selected each response option 50% of the time. Differences between conditions were analyzed with Bayes factor analyses that quantify the strength of the evidence in favor of the null versus alternative hypotheses (Rouder, Speckman, Sun, Morey, & Iverson, 2009).
2. By quantifying the feature tuning of serial dependence. For each trial, response error was the distance between response and current shape, and relative shape was the difference between current and previous trials (in morph units). The average response error was calculated for bins of 0.1 morph units of relative shape on individual data and then averaged across participants. This average response error was plotted against relative shape and fit with a derivative of Gaussian (DoG), which is given by *y* = *h* + (*x* + *b*)*awce* − (*w*(*x* + *b*))^2^, where *x* is the relative shape between successive trials, *a* is the amplitude of the curve, *w* is its width, *h* is its height, *b* is the intercept, and *c* the constant √2/*e*^−0.5^. The half-amplitude of the DoG is the measure of serial dependence. To ascertain significance, permutation tests were performed in which the *x*-labels (relative shape) were randomly shuffled between trials, and a new DoG was fitted on the shuffled data. This is equivalent to randomly shuffling the labels between the observed data and a null distribution of no serial dependence that has the same biases as the empirical data (parameters *b* and *h*). This was done 1000 times. Conditions with 95% confidence intervals that exclude zero were considered significant.
3. As a percentage by considering error trials (i.e., trials in which the shape value was less than 0.5 in which observers reported seeing a square and trials in which the shape value was greater than 0.5 in which observers reported seeing a circle). The number of trials in which the response erred toward the previously presented shape was divided by the total number of errors. If there was no relationship between current response and previous shape, 50% of the errors should be preceded by more circle-like shapes and 50% by more square-like shapes. For ease of interpretation, 50% was thus subtracted from the percent serial dependence. Thus, no serial dependence was 0%, 50% serial dependence was if all erroneous responses erred toward the previous shape, and −50% serial dependence was if all erroneous responses erred away from the previous shape (a repulsive effect). This analysis was performed on individual data. Statistical significance was ascertained by *t*-tests and, in Experiment 2, by a linear mixed model (LMM) with previous and current responses as fixed effects (both with two levels: report one of two shapes vs. report the ensemble) and random intercepts. Significant effects were those with |*t*| > 2. Non-significant effects were further examined with Bayes factors.

### Results

#### Experiment 1

**Figure 2.**
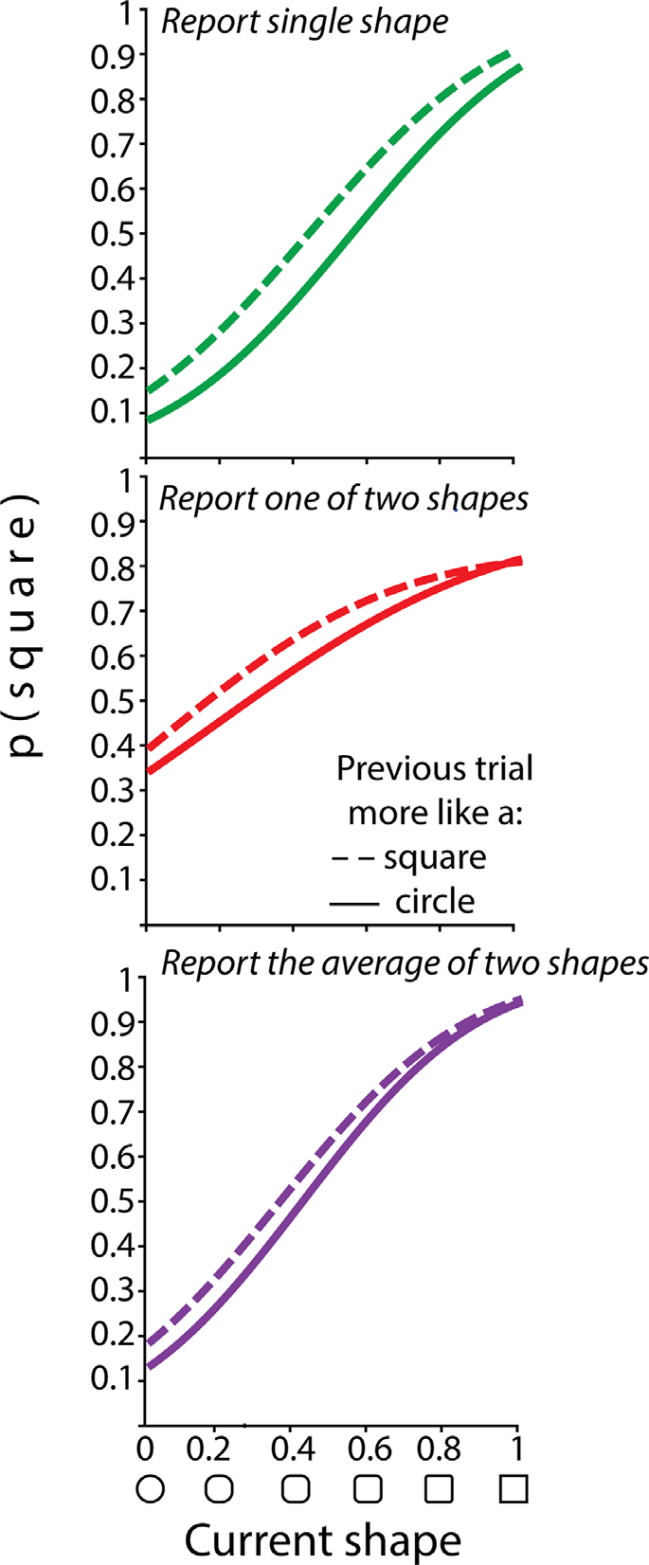
Psychometric functions (probability of responding “square” as a function of physical shape value, in morph units). (Top panel) In Experiment 1, when participants reported the shape of the single item. (Middle panel) In Experiment 1, when participants had to report one of two items. (Bottom panel) In Experiment 2, when participants had to report the average of two shapes (ensemble response).

Figure 2 shows psychometric functions, with trials divided depending on whether they were preceded by a more square-like shape or a more circle-like shape. Serial dependence can be seen in the leftward shift of the psychometric function when the previous trial was more like a square relative to the psychometric function when the previous trial was more like a circle. When participants had to report a single shape (top panel), the alternative hypothesis of a difference of PSEs between trials preceded by a more square-like shape and those preceded by a more circle-like was 8.33 times more likely than the null (absence of difference). Using the scale of interpretation suggested by Lee and Wagenmakers (2013), this evidence can be qualitatively labeled as “moderate.” Recall that the qualitative labels for evidence given by Bayes factors, which Lee and Wagenmakers (2013) updated from the original Jeffreys scale, are, in order, “barely worth more than a mention,” “anecdotal,” “moderate,” “strong,” “very strong,” “decisive,” and “extreme.” When participants had to report one of two shapes (middle panel), the alternative of a difference between the two PSEs was 3.76 more likely than the null of no difference (again, “moderate” evidence against the null).

**Figure 3.**
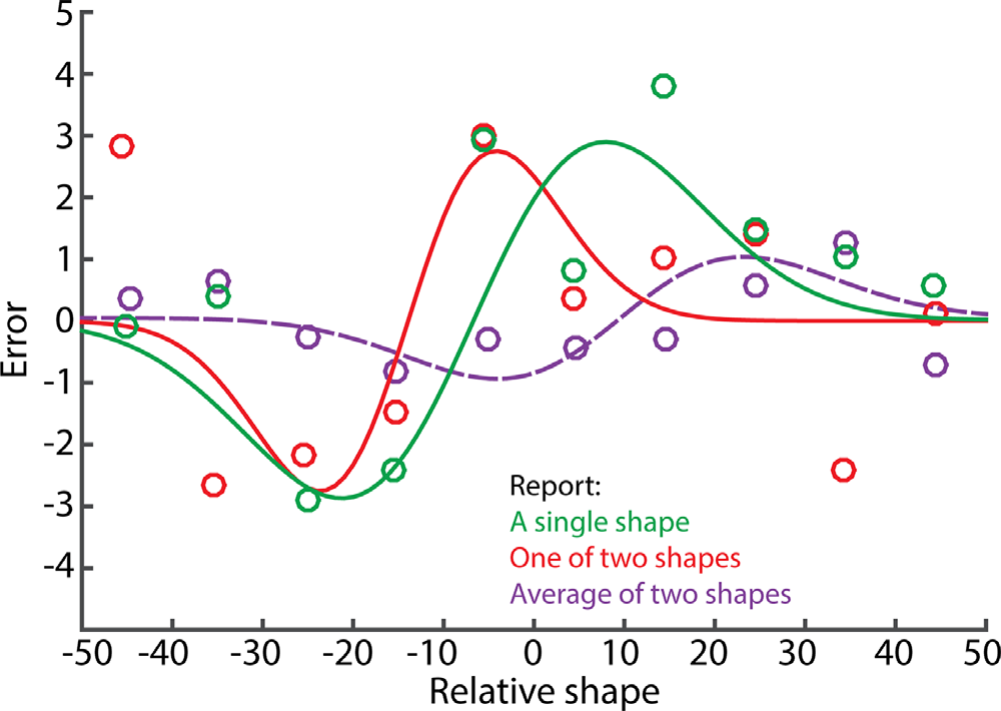
Feature tuning of serial dependence. When participants reported the single shape (green), response error was reported as a function of relative shape between previous and current shapes. When participants reported one of two shapes (red), response error was reported as a function of relative shape between previous and current shapes on the same side. When participants reported the ensemble (dashed purple), response error was reported as a function of relative shape between previous and current ensembles.

Figure 3 shows the feature tuning of serial dependence. The amplitude of the DoG fit was significant both when participants had to report a single shape (3.1; 95% confidence interval [CI], 1.4–4.7; green curve) and when they had to report one of two shapes (2.5; 95% CI, 0.6–2.9; red curve).

**Figure 4.**
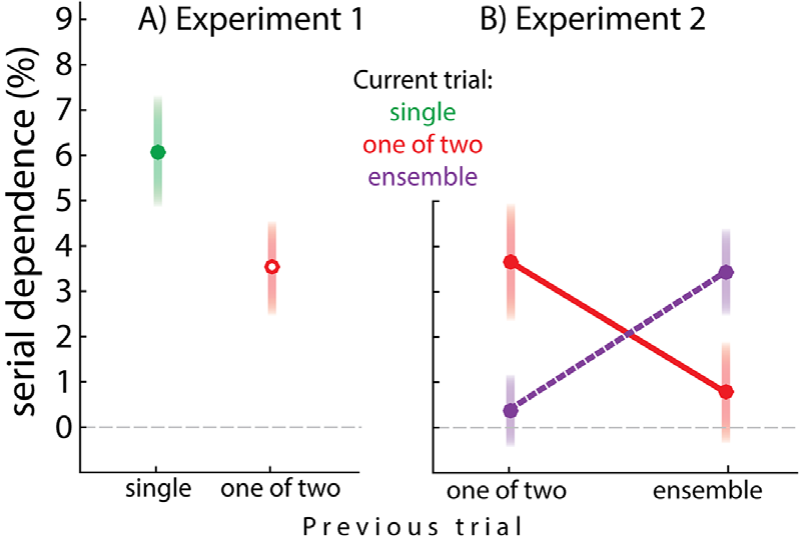
Percent serial dependence as a function of what had been reported in the previous trial and what was reported in the current trial (a single shape, one of two shapes, or the ensemble). Error bars represent *SEM*. (A) Experiment 1. (B) Experiment 2.

Figure 4 quantifies serial dependence as a percentage, as a function of what participants reported on the current trial and what they had reported on the previous trial (a single shape or one of two shapes). Perception of single shapes depended on the immediately preceding shape: mean ± *SEM*, 6.1% ± 1.2%; *t*(14) = 4.9; *p* < 0.0002 (Figure 4A, filled green symbol and bar). Serial dependence was also observed between individual shapes on the same side when two shapes were present: 3.5% ± 1.0%; *t*(14) = 3.2; *p* < 0.007 (Figure 4A, open red symbol and bar).

These analyses establish that the shape stimuli used in the current experiment are subject to serial dependence. In addition, they show that the preceding shape on the same side influenced current perception regardless of whether or not that shape had been cued: cued, 3.3% ± 1.4%; uncued, 3.8% ± 1.6%; both significantly greater zero, *t*(14) = 2.2, *p* < 0.021 and *t*(14) = 2.3, *p* < 0.019, but not significantly different from each other, *t*(14) < 1, *BF* = 0.27. Furthermore, the serial dependence of current response on previously cued shape was not significantly different from zero when the previously cued shape was at the opposite location: 0.6% + 1.1%; *t*(14) < 1; *BF* = 3.0. These results suggest that each object (or side) is monitored independently.

Finally, although in Experiment 1 participants were never asked to report the average shape, it is nevertheless possible to examine whether current perception is pulled toward the previous ensemble. There was no significant serial dependence of current shape on previous ensemble: 2.3% ± 2.0%; *t*(14) = 1.1; *p* > 0.25; *BF* = 0.46.

#### Experiment 2

The bottom panel of Figure 2 shows psychometric functions for trials in which participants had to report the average of two shapes (i.e., an ensemble response). Serial dependence can again be seen in the shift between the two functions, with more “square” responses when the preceding trial was more like a square than when it was more like a circle. The hypothesis of a difference of PSEs was 3.55 times more likely than the null hypothesis of no difference (“moderate” evidence against the null).

The purple dashed line in Figure 3 shows feature tuning for the serial dependence of ensembles, calculated as in Experiment 1. The amplitude of the DoG was significant (0.79; 95% CI, 0.38–1.14).

The main analysis of this study is on double-shape and ensemble trials. If ensemble perception is obligatory, then perception of individual shapes should be pulled toward the previous ensemble. Alternatively, if ensemble perception is not obligatory, then the perception of an individual shape would depend on the previous individual shape at that location. This would suggest that serial dependence occurs simultaneously for different stimuli: individual items and the ensemble representation of those items.

The interaction illustrated in Figure 4B reveals that the perception of individual shapes and ensembles depended on the previously seen individual shape or ensemble, respectively. The interaction term in the LMM was significant (*t* = 3.0). When asked to report an individual shape, perception was pulled toward the previously reported shape at the same location. The amount of serial dependence was 3.7% ± 1.3% (*t* = 3.5). Less serial dependence occurred between individual shapes and the previously reported ensemble (0.8% ± 1.1%; *t* = −2.72). In fact, the amount of serial dependence of perceived individual shapes on previous ensembles failed to reach significance (*t* = 0.73; *BF* = 0.30). However, individual shapes preceded by ensembles were attracted toward the previously non-reported shape on the same side: 2.2 ± 0.7; *t*(18) = 3.0; *p* < 0.009.

When asked to report the ensemble, perception depended on the previously reported ensemble (3.4% ± 1.0%; *t* = 3.2) and less on the previously cued individual shape (0.3% ± 0.8%; *t* = −2.17). In fact, serial dependence of perceived ensembles on previously cued individual shapes failed to reach significance (*t* = 0.33; *BF* = 0.26). Interestingly, when the previous ensemble had not been cued for report (i.e., participants had reported one of the two previous objects), serial dependence of ensemble perception on the current trial failed to reach significance: 1.9% ± 1.1%; *t*(18) = 1.58; *p* > 0.13; *BF* = 0.29. This replicates the result found in Experiment 1.

## Discussion

When participants were asked to report the shape of an individual object, their responses were attracted toward the shape of that object seen in the recent past. They were not influenced by a shape on the opposite side, even when that shape had been previously cued for report. This serial dependence between objects at the same location, but not across locations, may seem to contrast with results from other studies showing that serial dependence operates across locations (if they are not too far apart) (Collins, 2019). However, in these previous studies, there was only one object, compared with the two in the current experiment. Thus, individual objects within multi-object scenes may be monitored by independent continuity fields (Fischer, Czoschke, Peters, Rahm, Kaiser, & Bledowski, 2020).

When participants were asked to report the shape of an individual object, their responses were not attracted to the previous ensemble, even when they had reported that ensemble. This result suggests that there are two independent representations, one for the individual object and the other for the ensemble. This condition extends previous studies of serial dependence with ensemble stimuli, in particular (Manassi et al., 2017). Indeed, in that study, trials alternated between one Gabor (participants had to report the single orientation) and nine Gabors (participants had to report the average orientation). There was serial dependence of the single Gabor on the previous ensemble, as well as serial dependence of the ensemble on the previous single Gabor. This does not test if ensemble representation is obligatory, as there was no condition with an ensemble of Gabors on which subjects were not asked report the average. To test whether ensemble perception is obligatory, it is necessary to include trials on which an ensemble of items is presented but participants report only one of the items. The current study thus made it possible to determine whether the perception of an individual item on a given trial is influenced by the previous ensemble (it is not) or the previous individual item (it is).

When participants reported the average of two shapes, they were influenced by the previous ensemble representation, replicating both Manassi et al. (2017) and Pascucci et al. (2019). This attractive influence of the previous ensemble on the current ensemble occurred only when participants had previously reported that ensemble. When they had reported an individual object in the previous trial, the non-reported ensemble did not influence subsequent perception.

When viewing a multi-object scene, individual object features are stored, possibly in an object-file-like representation, and integrated with features from previous samples of that object. This integrated representation then gives rise to current perception. It is not necessary to pay attention to the individual object for its features to be integrated into the object file (assuming that post-cueing for report orients attention to a memory trace). Serial dependence of individual object perception on previous individual objects occurred both in trials in which participants had reported the individual object and in trials in which they had reported the ensemble. When viewing a multi-object scene, ensemble representations are stored only when attention is directed to the ensemble, as shown by the presence of serial dependence between ensemble trials, but no influence of *non*-reported ensembles on current ensembles. These results suggest that, although the perception of individual objects is obligatory, ensemble perception is not. They furthermore suggest that individual object features can contribute to different object files at the same time: the file corresponding to the individual object and, if attention is drawn to the ensemble of which the object is a part, to the ensemble file. Thus, individual object representations and ensemble representations can be maintained, and integrated with previous history, in parallel. In other words, multiple continuity fields operate simultaneously on object and scene representations.
